# Supplementary material for: Comparison of Antimicrobial Resistances in Escherichia coli from Conventionally and Organic Farmed Poultry from Germany
Source: Antibiotics (Basel). 2022 Sep 21;11(10):1282. doi: 10.3390/antibiotics11101282 (PMC9598375; doi:10.3390/antibiotics11101282)
Supplement: Supplementary file 1 [file antibiotics-11-01282-s001.zip › Table S1.pdf]

Table S1. Association of resistance with production system and matrix using logistic regression*

Substance	Category	p-value	Odds ratio	Lower 95% C.I. for EXP(B)	Upper 95% C.I. for EXP(B)
Ampicillin	org_conv	<.001	3.62712	2.542	5.175
	Source	.625			
	Turkey at farm	.512	.887	.620	1.269
	Turkey meat	.351	.850	.604	1.196
	Constant	<.001	2.211		
Azithromycin	org_conv	.294	3.074	.378	25.037
	Source	.438			
	Turkey at farm	.336	1.802	.543	5.982
	Turkey meat	.740	.779	.178	3.405
	Constant	<.001	.016		
Chloraphenicol	org_conv	.002	2.385	1.370	4.153
	Source	<.001			
	Turkey at farm	<.001	3.183	1.856	5.459
	Turkey meat	<.001	3.57	2.104	6.057
	Constant	<.001	.079		
Ciprofloxacin	org_conv	<.001	3.346	2.163	5.177
	Source	.112			
	Turkey at farm	.058	.709	.497	1.012
	Turkey meat	.116	.758	.537	1.071
	Constant	.023	.773		
Colistin	org_conv	.049	2.315	1.004	5.338
	Source	.969			
	Turkey at farm	.839	1.068	.568	2.008
	Turkey meat	.962	.985	.523	1.854
	Constant	<.001	.086		
Cefotaxime	org_conv	.491	.690	.240	1.983
	Source	.713			
	Turkey at farm	.575	1.431	.409	5.006
	Turkey meat	.418	1.621	.503	5.222
	Constant	.000	.021		
Gentamicin	org_conv	.188	1.802	.749	4.334
	Source	.005			
	Turkey at farm	.046	3.027	1.020	8.982
	Turkey meat	.001	5.243	1.910	14.388
	Constant	<.001	.016		
Nalidixic acid	org_conv	<.001	2.683	1.664	4.325
	Source	<.001			
	Turkey at farm	<.001	.451	.307	.664
	Turkey meat	<.001	.485	.335	.702
	Constant	.001	.679		
Sulfamethoxazole	org_conv	<.001	4.272	2.778	6.568
	Source	<.001			
	Turkey at farm	<.001	.435	.305	.620
	Turkey meat	<.001	.546	.389	.766
	Constant	.007	1.361		

Ceftazidime	org_conv	.357	.580	.182	1.847
	Source	.820			
	Turkey at farm	.585	1.418	.405	4.966
	Turkey meat	.997	1.003	.279	3.603
	Constant	<.001	.014		
Tetracyclin	org_conv	<.001	4.000	2.689	5.950
	Source	<.001			
	Turkey at farm	.042	1.436	1.013	2.033
	Turkey meat	<.001	2.052	1.457	2.890
	Constant	<.001	.659		
Trimethoprim	org_conv	<.001	5.048	3.013	8.458
	Source	<.001			
	Turkey at farm	<.001	.301	.205	.442
	Turkey meat	<.001	.464	.326	.661
	Constant	.627	1.057		
Fully susceptible	org_conv	<.001	.191	.132	.277
	Source	.060			
	Turkey at farm	.018	1.65	1.091	2.495
	Turkey meat	.264	1.256	.842	1.875
	Constant	.862	.963		
Multiresistant	org_conv	<.001	4.122	2.691	6.313
	Source	.505			
	Turkey at farm	.247	.814	.575	1.153
	Turkey meat	.533	.898	.640	1.260
	Constant	.000	.218		

* Meropenem and tigecycline were not included in the analysis as no resistant isolates were found. Broilers at farm and conventional were the reference categories.

conv = conventional farming

org = organic farming
