# Supplementary material for: Comparison of Antimicrobial Resistances in Escherichia coli from Conventionally and Organic Farmed Poultry from Germany
Source: Antibiotics (Basel). 2022 Sep 21;11(10):1282. doi: 10.3390/antibiotics11101282 (PMC9598375; doi:10.3390/antibiotics11101282)
Supplement: Supplementary file 1 [file antibiotics-11-01282-s001.zip › Table S2.pdf]

Table S2 Comparison of resistance of *E. coli* isolates from organic and conventional husbandry calculated for each of the three different categories.*

Substance	Category		p-value	Odds ratio	Lower 95% C.I. for EXP(B)	Upper 95% C.I. for EXP(B)
Ampicillin	Turkey at farm	org_conv	.009	2.848	1.297	6.253
		Constant	.277	.667		
	Turkey meat	org_conv	<.001	3.054	1.935	4.821
		Constant	.002	.576		
	Broiler at farm	org_conv	<.001	8.167	3.396	19.642
		Constant	.004	.292		
Azithromycin	Turkey at farm	org_conv	.790	.744	.084	6.592
		Constant	.001	.034		
	Turkey meat [a]					
	Broiler at farm [a]					
Chloramphenicol	Turkey at farm	org_conv	.201	2.250	.650	7.791
		Constant	<.001	.111		
	Turkey meat	org_conv	.013	2.230	1.182	4.206
		Constant	<.001	.126		
	Broiler at farm	org_conv	.998	133654225.888	<.001	.
		Constant	.998	<.001		
Ciprofloxacin	Turkey at farm	org_conv	.053	2.692	.987	7.342
		Constant	.001	.200		
	Turkey meat	org_conv	<.001	2.828	1.644	4.865
		Constant	<.001	.196		
	Broiler at farm	org_conv	.001	7.489	2.228	25.169
		Constant	<.001	.107		
Colistin	Turkey at farm [a]					
	Turkey meat	org_conv	.599	1.289	.5	3.32
		Constant	<.001	.055		
	Broiler at farm [a]					
Cefotaxime	Turkey at farm	org_conv	.098	.213	.034	1.332
		Constant	<.001	.071		
	Turkey meat	org_conv	.812	.851	.224	3.228
		Constant	<.001	.031		
	Broiler at farm [a]					
Gentamicin	Turkey at farm [a]					
	Turkey meat	org_conv	.251	1.768	.668	4.68
		Constant	<.001	.047		
	Broiler at farm	org_conv	.424	.404	.044	3.732
		Constant	.001	.033		
Nalidixic acid	Turkey at farm	org_conv	.238	1.942	.644	5.85
		Constant	<.001	.154		
	Turkey meat	org_conv	.016	2.111	1.151	3.871

		Constant	<.001	.145		
	Broiler at farm	org_conv	.002	6.629	1.972	22.286
		Constant	<.001	.107		
Sulfamethoxazole	Turkey at farm	org_conv	.091	2.250	.879	5.76
		Constant	.002	.250		
	Turkey meat	org_conv	<.001	3.662	2.138	6.271
		Constant	<.001	.196		
	Broiler at farm	org_conv	<.001	13.507	4.017	45.414
		Constant	<.001	.107		
**C**eftazidime	Turkey at farm	org_conv	.098	.213	.034	1.332
		Constant	<.001	.071		
	Turkey meat	org_conv	.638	.679	.135	3.415
		Constant	<.001	.023		
	Broiler at farm [a]					
Tetracycline	Turkey at farm	org_conv	.002	4.804	1.768	13.051
		Constant	.001	.200		
	Turkey meat	org_conv	<.001	3.555	2.211	5.717
		Constant	<.001	.367		
	Broiler at farm	org_conv	.003	6.274	1.866	21.098
		Constant	<.001	.107		
Trimethoprim	Turkey at farm	org_conv	.238	1.942	.644	5.85
		Constant	<.001	.154		
	Turkey meat	org_conv	<.001	4.409	2.311	8.41
		Constant	<.001	.107		
	Broiler at farm	org_conv	<.001	16.021	3.756	68.343
		Constant	<.001	.069		
Fully susceptible	Turkey at farm	org_conv	.002	.284	.129	.623
		Constant	.715	1.143		
	Turkey meat	org_conv	<.001	.249	.154	.404
		Constant	.730	1.062		
	Broiler at farm	org_conv	<.001	.063	.027	.146
		Constant	.024	2.444		
Multiresistant	Turkey at farm	org_conv	.070	2.283	.936	5.57
		Constant	.006	.304		
	Turkey meat	org_conv	<.001	4.148	2.425	7.095
		Constant	<.001	.196		
	Broiler at farm	org_conv	<.001	8.675	2.582	29.149
		Constant	<.001	.107		

* Meropenem and tigecycline were not included in the analysis as no resistant isolates were found.

[a] For C.I 0 logistic regression was not performed (-)

conv = conventional farming

org = organic farming
